# Trajectories of Mental Health Problems in Childhood and Adult Voting Behaviour: Evidence from the 1970s British Cohort Study

**DOI:** 10.1007/s11109-022-09852-9

**Published:** 2023-01-21

**Authors:** Lisa-Christine Girard, Martin Okolikj

**Affiliations:** 1 School of Health in Social Science, Clinical Psychology, University of Edinburgh, Edinburgh, UK; 2 Present Address: Department of Psychosocial Science, University of Bergen, Bergen, Norway; 3 Department of Comparative Politics, University of Bergen, Bergen, Norway

**Keywords:** Mental health, Conduct problems, Voter turnout, 1970 British Cohort Study

## Abstract

The link between childhood mental health difficulties such as conduct problems and adult political abstention has been overlooked despite early mental health difficulties potentially resulting in political self-marginalisation. Using the1970s British Cohort Study, we estimate developmental trajectories of conduct problems (i.e., from 5 to 16 years). Logistic regression, linear probability models, and propensity score matching were then conducted to test the association between trajectory group membership and voter turnout at 30, 42, and 46 years of age. Three distinct trajectories of conduct problems were identified: a normative (*n* = 11,871; reference group), moderate-chronic (*n* = 3433), and elevated-chronic (*n* = 250) group. Results revealed an association between conduct problems and decreased turnout. In particular the elevated-chronic group had a decreased odds of voting of 52.2%, 52.0%, and 45.7%, as compared to the normative group at 30, 42, and 46 years respectively. The moderate-chronic group had a decreased odds of voting of 24.7% as compared to the normative group at age 30 only. Matched results and linear probability models substantiated findings, suggesting (1) the importance of considering childhood factors when examining antecedents of lifelong voting behaviour, and (2) the political self-marginalisation of people with chronic childhood conduct problems more than 3 decades later.

**Supplementary Information:**

The online version contains supplementary material available at 10.1007/s11109-022-09852-9.

## Introduction

A call for action to better address the Mental Health Burden has been ongoing since the new millennium (World Health Organization, 2001). Urgency has been placed on ensuring mental health (MH) is a priority for health and development agendas at both the national and international level. In the wake of COVID-19, this urgency is even more critical as reported estimates of MH difficulties have increased steadily at alarming rates, particularly in children and adolescents, which has been likened to a ‘psychiatric epidemic’ (Hossain et al., 2020). The fall out with higher incidence of maladaptive outcomes associated with these rising MH difficulties will likely be disastrous in the years to come. At the same time, the quality of democracy and the democratic legitimacy of elected governments have been brought into question due to the observed declining turnout rates. The crisis of democratic legitimacy has substantially deepened since the 2007–2008 Global Financial Crisis (Kriesi, 2013), and in line with the current COVID-19 pandemic, this downward trend of democratic legitimacy is likely to continue. Thus, whilst not new, the question of who abstains from voting remains of growing importance.

Indeed, extensive work has been carried out demonstrating that marginalised groups (e.g., racial and ethnic minorities, people with lower SES) are amongst the least likely to vote (Dalton, 2017; Fraga, 2018; Simonsen, 2021). Arguably, people with MH difficulties also constitute a marginalised group given the long-standing discourse of stigmatisation and discrimination that has only recently begun to change. And yet, research examining the link between MH and voting is still in its infancy. Now more than ever it is imperative to better understand how citizen’s MH, especially starting in early childhood, may translate into becoming part of an underrepresented and marginalised group within democratic societies across the lifespan. Particularly so given that nearly half of all MH difficulties emerge before 14 years of age (World Health Organisation, 2021). Moreover, this is important given that studies have shown political maturity and voting habits to already be established by adolescence, prior to the age of 16 (Okolikj & Hooghe, 2022).

The most common MH presentations in childhood and adolescence include emotional (e.g., depression, anxiety) and behavioural difficulties (e.g., conduct problems) (e.g., Ford et al., 2003), which are commonly conceptualised as forms of internalising and externalising difficulties, respectively. Internalising and externalising difficulties are distinct from each other, although heterotypic comorbidity has been found across development (e.g., Girard, 2021). Global estimates suggest up to 20% of children and adolescents are affected by these MH difficulties, with conduct problems (classified as an externalising difficulty) being amongst one of the leading causes of the MH burden in this age group (World Health Organization, 2020).

### Conduct Problems

Conduct problems are characterized by aggressive and non-aggressive behaviours, that repeatedly violate age-appropriate social norms and/or the rights of others, in children and adolescents 18 years of age and under (American Psychiatric Association, 2013). Some examples of behavioural characteristics typical of children and adolescents exhibiting conduct problems would include repetitive and continual physical aggressions towards others, starting fights, bullying or intimidating others, destruction of property, lying and deceitfulness to obtain their own needs, theft, running away from home, and truancy from school. There is vast heterogeneity in the presentation of symptom severity with a spectrum ranging between conduct *problems* to conduct *disorder* (Barry et al., 2013). Moreover, age of onset is a distinguishing diagnostic feature with both early-onset and adolescent-onset conduct problems. Early-onset and persistent conduct problems have been reported as affecting between 5 and 7% of children globally (Ghandour et al., 2019; Rivenbark et al., 2018), and in some developed countries it is estimated that conduct problems account for as high as 50% of MH referrals (Center for Mental Health Services, 2010). However, not all children with early-onset conduct problems will continue with chronic conduct problems, and many with adolescent-onset will remit with time. As conduct problems are a developmental MH problem, continuation of these behavioural characteristics on the severe end of the spectrum into adulthood becomes classified as antisocial personality disorder.

Developmental and economic research has shown that impairment resulting from the presence of early conduct problems across multiple domains of living is vast and extends beyond the childhood and adolescence period, into adulthood. For example, early conduct problems have been associated with a host of maladaptive outcomes in adulthood, including lower educational and occupational attainment, lower wages, increased criminality, incarcerations, hospitalisations, MH difficulties (e.g., antisocial personality disorder), suicide attempts, homelessness, and substance abuse amongst others (Bevilacqua et al., 2018; Fergusson et al., 2005; Heckman, 2011; Moffit et al., 2002; Odgers et al., 2008; Rivenbark et al., 2018). Moreover, early stigmatization faced by children with conduct problems can carry with it a heavy psychological burden into adulthood. Together, this results in high personal and societal costs through increased reliance on public services across sectors (health, social, justice system; see Rivenbark et al., 2018). Cost analysis reveals individual costs of early-onset conduct problems from childhood to preteen at $70,000 annually and increased service use (3.5 times higher for conduct problems, 10 times higher for conduct disorder) in adulthood (Scott et al., 2001). This ensuing cost for society is particularly important given the rise in prevalence rates of conduct problems in childhood and adolescence since the mid-late twentieth century (Collishaw et al., 2004).

Noteworthy, political abstention as an outcome of early conduct problems, which carries with it a high cost not only for individuals with conduct problems but also society in general, has been seemingly overlooked. This link between political participation and conduct problems may have serious consequences for those with early conduct problems. For example, political self-marginalisation in the democratic process negates the possibility of implementation of policies specific to the improvement of social status. Second, early predictors of participation emerging in childhood warrant particular attention given: (1) this is a malleable period and (2) their potential to create political inequalities in adulthood. Given the nature of conduct problems and the associated long term maladaptive outcomes, with high individual and societal costs, it is imperative that we better understand the potential path towards political disengagement and ensuing likelihood for political and representational policy inequalities.

### Mental Health and Voter Turnout

Interestingly, it has only been over the past decade that studies have examined the role of MH on voter turnout, focusing either on overall MH (broadly defined), specific internalising difficulties (e.g., depression) starting in late adolescence/adulthood, or MH anchored within but not clearly differentiated from ‘disability’. Indeed, existing negative associations between MH and voter turnout have emerged (e.g., Couture & Breux, 2017; Denny & Doyle, 2007; Landwehr & Ojeda, 2021; Mattila & Papageorgiou, 2017; Ojeda, 2015; Reher, 2020; Wray-Lake et al., 2019), substantially advancing current knowledge by identifying that citizens suffering from certain MH difficulties are a self-marginalised group when it comes to elections. Here, it has been argued that the link between overall MH and turnout results from the perceived high cost (i.e., effort) outweighing the benefit (e.g., Denny & Doyle, 2008), and in the case of depression, from lower motivation and cognitive abilities, which are required for political participation (Ojeda, 2015). These factors (motivation and cognitive abilities) in particular, may impose large costs on voting due to the anti-socialisation nature of their effects, reducing the civic skills required for voting. A recent large scale comparative study of depression and participation confirms this association cross nationally, finding that depressive symptoms lower the probability of voting between 5 and 25 points, whilst also showing negative effects on political motivation and political engagement (Landwehr & Ojeda, 2021). The institutional support, as it is the case with the disability rights movement, is largely missing for those with MH problems, resulting in a lack of institutional structure aimed to benefit and increase the political participation among those groups.

As mentioned, these studies have made important contributions by identifying a link between MH difficulties and voter turnout. However, we have only begun to scratch the surface of our understanding. Current gaps in the literature must also be noted. For example, externalising MH difficulties, early developmental periods, and chronicity have largely remained out of this literature’s spotlight. Externalising MH difficulties such as conduct problems (one of the leading causes of childhood MH difficulties), are associated with outcomes in adulthood that may preclude the likelihood of voting (e.g., substance abuse, hospitalisations, incarceration). Moreover, part of the very nature of what characterises conduct problems (i.e., the repetitive violation of social norms) is likely to be an early indicator that civic attitudes and actions are not at the forefront of these individuals’ thinking or behavioural repertoires. Despite this potential link, conduct problems in studies of turnout have not yet been examined. This is perhaps due to the greater attention on more proximal MH difficulties in late adolescence and adulthood (i.e., conduct problems are a developmental MH problem in children and adolescents under 18 years of age). However, in not examining conduct problems we potentially neglect up to 7% of the population amongst whom may be contributing to the declining turnout rates, consequently warranting attention.

MH difficulties in childhood are also presently overlooked in the turnout literature. Not examining MH in childhood and its consequences on adult turnout ignores a critical developmental period in the life-course when investment towards malleability of future outcomes is possible. This is reaffirmed in the ‘impressionable years hypothesis’ in political socialisation (Greenstein, 1965; Jennings & Niemi, 1968a, 1968b; Prior, 2010). Additionally, no studies to the best of our knowledge, have looked at chronicity of MH using a trajectory approach. This would allow a better understanding of the association as compared to examining MH at one point in time only. For example, modelling trajectories of MH provides the unique opportunity to identify distinct groups of individuals following similar developmental progressions over time (e.g., chronic, declining, early increasing later decreasing), and the potential nuanced effects of chronicity on turnout.

The construct of MH includes multiple domains (e.g., internalising and externalising), which evidence great heterogeneity not only in terms of etiology, but also in terms of associated outcomes. Thus, acknowledging and subsequently distinguishing between the independent roles of internalising and externalising types of MH difficulties is critical for furthering our understanding into the link with turnout. Currently, internalising MH difficulties have been the focal point. Thus, in this study we shift focus and begin to explore the role of an externalising MH difficulty for voter turnout. To the best of our knowledge, no studies examining MH and turnout have focused on childhood trajectories of conduct problems, which track stability and/or patterns of change of conduct problems from childhood to adolescence, whilst linking to voter turnout across differing stages in adulthood (i.e., using a lifespan approach). This represents a critical gap in the current knowledge base.

## Theoretical Expectations and Hypothesis

We argue that an association between early and sustained conduct problems and decreased voter turnout in adulthood could theoretically be driven by the following. First, via cognitive ability. Voting is argued to be a skills-based act (Brady et al., 1995), requiring higher cognitive functioning (both general and language specific) for understanding and engaging with parties/platforms, and for being able to express oneself politically (Brady et al., 1995; Denny & Doyle, 2008). Within the resource model of turnout, cognitive abilities have been recognised as a leading factor in predicting electoral participation (e.g., Denny & Doyle, 2008). Both general and language specific domains of cognition have been found to be underdeveloped in those with conduct problems (e.g., Fairchild et al., 2019; Girard et al., 2016), and this may result in a lower propensity for turnout in this group. The second theoretical path is via non-cognitive abilities, which includes self-regulation (e.g., delayed gratification, behavioural and effortful control) and interpersonal skills (e.g., empathy). Non-cognitive skills have more recently been paid closer attention to in the turnout literature, with demonstrated associations between poorer non-cognitive abilities and turnout, particularly amongst young voters (e.g., Holbein, 2017; Holbein & Hillygus, 2020). Deficits in non-cognitive skills are also particularly prevalent and characteristic of early-onset conduct problems (e.g., Fairchild et al., 2019), which may suggest another potential mechanism underlying the association.

Third, a key defining characteristic of conduct problems as stated in the Diagnostic and Statistical Manual IV (1994) is the repetitive violation of social rules and norms. Arguably, this routine engagement in rule breaking and violation of social norms could result in a lower propensity for high civic engagement and/or sense of civic responsibility over time (i.e., ‘civic duty’), including activities such as voting. If there is an early pattern of rule breaking owing to a low sense of responsibility towards society, it stands to reason that voting may not be an activity high on the priority list. This maps onto the work of Blais and Young (1999) suggesting that one’s sense of ‘duty’ to vote is one of the single strongest individual predictors of turnout.

Fourth, the association may result from poorer social capital amongst those with conduct problems. Dimensions of social capital of particular relevance include social networks, shared norms, and social participation (Harper, 2002). For example, the developmental and criminology literature suggests that children with conduct problems associate with peers who also engage in antisocial and rule breaking behaviours (i.e., social network) (Quinton, et al., 1993; Patterson et al., 2005), which may reinforce a normalisation of behaviours (i.e., shared norms). This group influence may result in the escalation of behaviours over time, leading to outcomes such as criminal convictions and imprisonment, coupled with a likely decrease in social participation. Consequently, potential political interest may be low, whilst mistrust in government and social systems may increase over time. Moreover, the social networks and support dimension of social capital can be viewed in line with Paul Lazarsfeld’s work in the 1950’s of ‘primary groups’, and the sociological rooting of turnout (Berelson et al., 1986). Here, it was suggested that there was political homogeneity within primary groups, in part resulting from the shared social characteristics and values, which translate into political (non-)behaviours.

Finally, individuals with sustained childhood conduct problems may be less likely to be targeted by political groups during campaigning (e.g., targeted party manifestos, cold calling, campaign contributions, interest groups, trade unions) in adulthood due to poorer accessibility (e.g., more transient, hospitalisations, incarcerations, homelessness). This may result in decreased accessibility of political information and knowledge, consequently lending to increases in political apathy.

Notwithstanding these proposed theoretical underpinnings, there are currently no studies that have examined the association between early and sustained conduct problems and adult voting behaviour, despite the potentially important implications (i.e., self-marginalised groups in democratic societies lead to a lack of inclusiveness and subsequent representation in the policy decision-making process). Thus, our primary aim was to examine whether childhood trajectories of conduct problems are associated with adult voter turnout.

### Hypothesis

Children in trajectory groups with elevated and chronic conduct problems will be less likely to vote in adulthood as compared to children without conduct problems or children with declining conduct problems. We expect results will hold after controlling for important family and individual-level factors, including political behaviours, more proximal mental health, and cognition.

## Data and Methods

Participants were drawn from the 1970s British Cohort Study, a longitudinal population-based birth cohort of ~ 17,000 infants born during the week of April 5th–11th, 1970, in Great Britain. Eight sweeps of follow-up data were collected between 1975 and 2016 (for full cohort details see Elliott & Shepherd, 2006). We use data from sweeps 1–6 (birth, five, 10, 16, 26, and 30 years of age), and 8–9 (42, and 46 years of age). Ethical approval was obtained using an internal ethical review prior to 2000 and a multicentre research ethics committee afterwards. Verbal consent was collected from all parents/guardians in the first 4 Sweeps and from cohort members in Sweeps 5–6, 8–9, prior to data collection.

### Outcome Measure

Voter turnout (yes/no) was collected using self-report at 46 years, asking participants whether they voted in the last general election. This fieldwork was conducted between July 2016 and July 2018. Halfway through the fieldwork, an early general election was held (June 2017). As a result, the question of turnout in the last election was with reference to the 2015 elections for participants asked prior to October 2017 (*n* = 4251), and to the 2017 election for participants asked after October 2017 (*n* = 4237). For the latter, they were also asked about the 2015 election as a recall question. Sensitivity analyses were conducted using turnout in 2015 only (supplementary material eTables 1–2), evidencing consistent findings. Given the uniqueness of the 2017 election (i.e., following BREXIT), and the potential for higher turnout in this specific election, we also examined voter turnout at age 30 and 42 years to ensure our findings were robust and not an artifact of the 2017 election. This also enabled us to examine the strength of association across differing periods of adulthood. When participants were 30, they were asked whether they voted in the last general election, in reference to the May 1997 election. At 42 years of age, participants were asked who they voted for in the last general election (in reference to the 2010 election), with a given list of all parties and an option ‘did not vote’. This latter information was then dichotomised to create a variable of voter turnout at age 42.

### Predictor and Controls

The parent-version of the Rutter A2 behaviour rating scale (Rutter et al., 1970) was used when participants were 5, 10 and 16 years old. The Rutter scale is a well validated screening tool completed by parents to flag potential childhood difficulties. A strength in using parent report is the breadth of knowledge regarding their own child’s behaviour across multiple settings. Parents rated their children on 18 behavioral items using a 3-point Likert scale, ranging from 1 (*does not apply*) to 3 (*certainly applies*). The scale was modified to a visual analogue scale ranging from 0 to 100, when children were 10 years of age. Details of the rescaling at age 10 are presented in the supplementary material (eFig. 1). Six items were used to capture children’s conduct problems: “often destroys own or others’ belongings”, “frequently fights other children”, “sometimes takes things belonging to others”, “bullies other children”, “often tells lies”, and “is often disobedient”. Reliability of the entire A2 scale has been demonstrated (i.e., inter-rater reliability, *r* = 0.64; retest reliability *r* = 0.74; see Rutter et al., 1970) and Cronbach’s alphas for conduct problems at five, 10 and 16 years in the current study were 0.72, 0.75, and 0.77, respectively.

Socio-demographic characteristics collected at birth included the participant’s sex (boy/girl), maternal age at first birth, family composition (married/single), parental social class (professional/managerial, non-manual/manual, unskilled/partly skilled, not working/other), region of birth (England, Wales, Scotland), and maternal age when left education. When participants were five, a test of visual spatial ability was conducted using the Copying Design Test (Davie et al., 1972). We use this measure as a proxy of children’s cognitive ability in our model. When participants were 26 years of age, information was collected regarding their highest level of education (less than high school/more than high school/not stated). At age 30, information was collected on participant’s political interest (not interested/interested/not stated), trade union membership (not a member/member/not stated), financial situation (living comfortably/doing alright/just about getting by/finding it quite difficult/finding it very difficult), and psychiatric morbidity using the Malaise Inventory (Rutter et al., 1970). The Malaise Inventory is comprised of 24 items covering psychological and somatic difficulties including depression, anxiety, and irritability. Example items include “do you often feel miserable or depressed”, “do you often get worried about things” and “do people often annoy and irritate you”, rated as 0 (*no*) or 1 (*yes*). Possible scores range from 0 to 24. High risk for psychiatric morbidity is considered present with a score above 7 (Rutter et al., 1976). At 42 years of age, information was again collected on participant’s highest level of education (less than high school/more than high school/not stated), political interest (not interested/interested/not stated), and trade union membership (not a member/member/not stated). Additional information was also collected regarding the participant’s social class (lower class/working class/lower middle class/middle class/upper middle class/upper class). At age 46, information was collected regarding the participant’s total household take home income after tax and deductions.^1^

### Statistical Analyses

We examine conduct problems from 5 to 16 years of age using group-based trajectory models (GBTM).^2^ Trajectories are the preferred modelling strategy as they capture the evolution of conduct problems across the ages examined (i.e., both intensity and growth). GBTM in particular is a person-centred approach, using finite mixture modelling to identify heterogeneity between groups of individuals following distinct developmental trajectories of conduct problems over time. This modelling approach differs from other trajectory approaches such as standard growth curve modelling, that start by examining individual variability around a mean-level trend. Instead, this person-centred approach is particularly well suited for modelling the evolution of a ‘non-normative’ behaviour such as conduct problems across development, as potential nuances of change and/or continuity for distinct groups of individuals following similar trajectories are well captured. Moreover, assumptions of linearity and homogeneity, which are common to variable-centred approaches in trajectory modelling (Bergman et al., 2015), are not imposed in GBTM. Both linear (e.g., increasing and decreasing) and quadratic growth can be estimated within this modelling strategy. When estimating trajectories, participants had to have at least one data point on conduct problems to be included in the model. This resulted in 15,554 participants being included in the trajectory analysis.

Censored-normed models, with a quasi-Newton procedure was used for estimating model parameters. To assess model fit, the Bayesian Information Criteria (BIC) and the Akaike Information Criterion (AIC) were used. In comparison to alternative trajectory approaches where a smaller BIC indicates good model fit, a larger more positive value of the BIC is indicative of a better model fit in GBTM (see Nagin, 2005; Raftery, 1995; Schwarz, 1978). However, the BIC and AIC alone cannot always properly assess model accuracy. Thus, the average posterior probabilities of group membership by trajectory membership group (APP), and the odds of correct classification (OCC) were also used in assessing the model fit, which were calculated post model estimation. The APP specifies the likelihood that a participant with a specific behavioural profile belongs to the model’s *J* trajectory group, with an upper bound of 1. The OCC indicates the odds that a participant has been correctly classified into trajectory group *j*, X times better than by chance alone (Nagin, 2005). Group assignment accuracy thresholds suggested for the APP and OCC are greater than 0.70 (i.e., 70%) and greater than 5, respectively (Nagin, 2005).

A two-stage approach was used in identifying the best model fit. First, we ran two-, three-, four-, five- and six-group models comparing the BIC and AIC of each model (eTable 3). We did not estimate models with larger than six-groups based on the principle of parsimony and the lack of improvement in the BIC and AIC between the five- and six-group model. Whilst the four and five-group models provided the best fit, the next step of fitting polynomial terms (both *linear* and *quadratic growth*) in these models revealed poor fit to the data (i.e., the identification of groups with no participants in the estimated trajectories). As a result, we fit polynomial terms (once again both linear and quadratic growth) in the three-group model, whose BIC was close to the four-group model. The three-group model with linear growth only, provided the best fit to this data, with post calculation of the APP and OCC further supporting the model (eTable 4). The three-group model was consequently selected.^3^

Logistic regression models were then estimated to examine the association between trajectories of conduct problems from five to 16 years of age and voter turnout at 30, 42 and 46 years of age, controlling for sweep 1 (birth), and sweep 2 (age five) covariates across all models. For the age 30 model, additional covariates collected at ages 26 and 30 (i.e., adult socio-demographics, psychiatric morbidity, and political behaviour) were included. For the age 42 model, the additional covariates included were collected when participants were age 30 and 42. In the age 46 model, the additional covariates used in the model were collected at ages 30, 42, and 46. Common in longitudinal studies, attrition and non-response across sweeps resulted in a reduced final sample of *n* = 6018, *n* = 5651, and *n* = 5396 at ages 30, 42, and 46, respectively. Linear Probability Models (LPM) were also conducted given their ease of interpretability, whereby the predicted value is the probability to vote. Robust standard errors were estimated to account for the inherent heteroscedasticity of the error term within the LPM approach. Given the possibility of post treatment bias in using variables collected following the estimation of trajectories, which may be causally affected by an individual’s conduct problems, we further ran logistic regression models using only birth and age 5 covariates as a sensitivity analysis. These models can be found in the supplementary material, eTable 5, which evidenced consistent findings.

Finally, given the evidence demonstrating the effect of early family characteristics and cognition on both conduct problems and voting behaviour, we use propensity score matching (PSM) as a further robustness check to determine whether the association would remain. The use of PSM helps to ensure equivalence between groups (e.g., elevated conduct problems, no conduct problems) by matching groups on the most relevant characteristics to reduce confounding. Comparisons between groups are then made, accounting for their measured characteristics and similar propensity for having conduct problems. While education level, social class, income, proximal mental health, political interest, and trade union membership may be strong predictors of voting turnout, their temporal order and lack of support to suggest an influence on conduct problems rendered their inclusion in the PSM model problematic. Thus, we include only the variables that precede our first assessment of conduct problems at age 5. We use nearest neighbour 1:1 modelling, with ties and a caliper, allowing for replacement. Nearest neighbour matching is conducted by first randomly ordering groups to ensure reduced bias during matching, as matching occurs sequentially. A common occurrence with categorical matching variables is multiple participants with identical propensity scores. Thus ties, which allow matching to multiple controls with identical propensity scores, are recommended (Abadie & Imbens, 2006). To ensure the most optimal matches between pairs, we further imposed a caliper of a fifth of a standard deviation. That is, for a match to occur, the propensity score of a matched pair had to fall within 0.05 of a standard deviation of one another. Matching with replacement was used given the low rates of participants with the most elevated level of conduct problems. Replacement reduces bias by ensuring that matches are of better quality (Caliendo & Kopeinig, 2008). Average treatment effects of those treated (ATT), is reported on. The statistical threshold in this study was set at p < 0.05, using two-tailed tests. Analyses were conducted in Stata v.17.0.

## Results

### Group-Based Trajectory Models

**Fig. 1:**
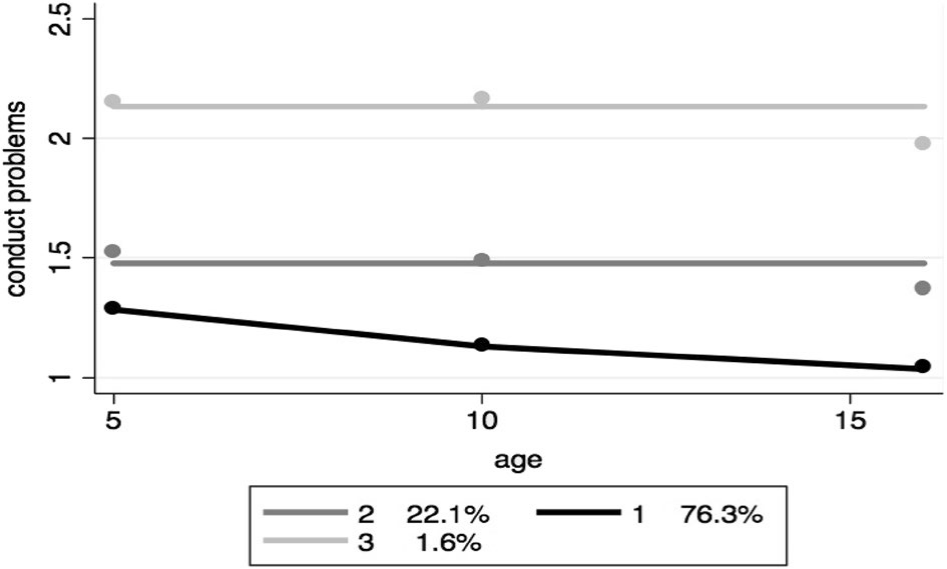
Group-based trajectories of conduct problems: From five to 16 years of age. *Note* Group 1 is labelled normative (n = 11,871), Group 2 is labelled moderate-chronic (n = 3433), Group 3 is labeled elevated-chronic (n = 250)

In total, 15,554 participants had data on conduct problems and were included in the trajectory models. Three distinct trajectories were identified. The first and largest group included an estimated 76.3% of the cohort, labelled ‘normative’. This group had low but slightly elevated conduct problems at age five, continually decreasing linearly across time, to non-existent levels between 10 and 16 years of age. Group 2, an estimated 22.1% of the cohort was labelled ‘moderate-chronic’. In this group, levels of conduct problems were higher than the normative group at age five but remained stable until 16 years of age. The final group, labelled ‘elevated-chronic’, consisted of an estimated 1.6% of the cohort. This group started with the highest levels of conduct problems at age five, remaining stable into adolescence (Fig. 1). Parameter estimates of trajectory groups are presented in Table 1. See eTables 6–8 for characteristics by groups at ages 30, 42, and 46.

**Table 1:** Trajectory parameter estimates for the 3-group model

Group	Parameter	Estimate	SE	*T*	*p*
Conduct problems					
1	Intercept	1.476	0.008	181.179	0.000
	Linear	− 0.052	0.001	− 45.451	0.000
2	Intercept	1.453	0.011	128.678	0.000
3	Intercept	2.134	0.032	66.337	0.000
	Sigma	0.388	0.002	156.101	0.000

*Note* Group 1 is normative (n = 11,871), Group 2 is moderate-chronic (n = 3,433), Group 3 is elevated-chronic (n = 250)

### Multivariable Analysis

To better understand whether group membership was associated with voter turnout when participants were 30, 42, and 46 years old, logistic regressions were conducted using the normative group as the reference category. Results revealed that trajectory group membership was associated with voter turnout, even after controlling for covariates. That is, participants in the elevated-chronic group had reduced *odds* of voting as compared to those in the normative group by 52.2%, 52.0% and 45.7%, at 30, 42, and 46 years of age, respectively. Similarly, participants in the moderate-chronic group had reduced *odds* of voting as compared to the normative group by 24.7% at 30 years of age, only (see Table 2). We further tested the association using LPM with robust standard errors. Results of these models substantiated findings whereby participants in the elevated-chronic group were 16.9, 15.9, and 13.5 percentage points less likely to vote as compared to the normative group at age 30, 42, and 46, respectively. In using LPM with robust standard errors, we also find that the moderate-chronic group were significantly less likely to vote as compared to the normative group across all ages (i.e., at 30, 42, and 46 years) by 6.3, 3.1, and 2.8 percentage points, respectively. Results of the LPM at age 46 are only significant at the 10% level. Full results of the LPM are presented in the supplementary material (eTable 9).

**Table 2:** Multivariable logistic regression model: conduct problems and voter turnout in the 2015/2017, 2010, and 1997 British General Elections

Variable	Turnout at 46			Turnout at 42			Turnout at 30		
	OR	*SE*	95% CI	OR	*SE*	95% CI	OR	*SE*	95% CI
Conduct Problems (ref. Normative)									
Moderate-chronic	0.846	(0.077)	[0.71–1.01]	0.853	(0.072)	[0.72–1.01]	0.753***	(0.055)	[0.65–0.87]
Elevated-chronic	0.543*	(0.158)	[0.31–0.96]	0.480*	(0.143)	[0.27–0.86]	0.478**	(0.130)	[0.28–0.82]
Sex (ref. Male)									
Female	1.571***	(0.121)	[1.35–1.83]	1.323***	(0.093)	[1.15–1.52]	1.315***	(0.077)	[1.17–1.48]
Education (ref. Less than high-school)									
More than high-school	1.975***	(0.181)	[1.65–2.36]	1.695***	(0.136)	[1.45–1.98]	1.113	(0.079)	[0.97–1.28]
Not stated	1.270	(0.228)	[0.89–1.80]				0.690**	(0.082)	[0.55–0.87]
Copy design (standardized)	1.036	(0.042)	[0.96–1.12]	1.037	(0.038)	[0.97–1.12]	0.982	(0.031)	[0.92–1.04]
Maternal age at first birth	1.028**	(0.011)	[1.01–1.05]	1.045***	(0.010)	[1.03–1.07]	1.014 +	(0.008)	[1.00–1.03]
Social class at birth (ref. managerial/professional)									
Non-manual/manual	0.922	(0.103)	[0.74–1.15]	0.882	(0.090)	[0.72–1.08]	1.005	(0.080)	[0.86–1.18]
Unskilled/partly-skilled	0.727*	(0.097)	[0.56–0.94]	0.843	(0.103)	[0.66–1.07]	0.880	(0.089)	[0.72–1.07]
Not working/other	0.542	(0.288)	[0.19–1.54]	0.494	(0.257)	[0.18–1.37]	0.545	(0.221)	[0.25–1.21]
Married (ref.)									
Single (includes: widowed, divorced, separated)	1.103	(0.199)	[0.78–1.57]	0.991	(0.155)	[0.73–1.35]	1.007	(0.149)	[0.75–1.35]
Maternal Age when Left Education	1.049^+^	(0.027)	[1.00–1.10]	1.065**	(0.025)	[1.02–1.16]	0.990	(0.172)	[0.96–1.02]
Trade Union (ref. not a member)									
Member	1.170	(0.135)	[0.93–1.47]	1.572***	(0.155)	[1.30–1.91]	1.413***	(0.101)	[1.23–1.63]
Not stated	0.954	(0.250)	[0.57–1.59]	1.954**	(0.482)	[1.20–3.17]			
Political Interest (ref. not interested)									
Interested	2.916***	(0.281)	[2.41–3.52]	4.712***	(0.392)	[4.00–5.55]	2.872***	(0.195)	[2.51–3.28]
Not stated	1.010	(0.283)	[0.58–1.75]	3.022 +	(1.97)	[0.84–10.8]			
Psychiatric morbidity (high)	0.783*	(0.086)	[0.63–0.97]	0.856	(0.088)	[0.70–1.05]	0.821*	(0.073)	[0.69–0.98]
Socio-economic-status									
Total income household	1.000*	(0.000)	[1.00–1.00]						
Social class				1.188***	(0.049)	[1.10–1.29]			
Current financial situation (reversed)							0.927*	(0.029)	[0.87–0.99]
Region of birth (ref. England)									
Wales	1.183	(0.200)	[0.85–1.65]	1.471*	(0.231)	[1.08–2.00]	1.471*	(0.188)	[1.15–1.89]
Scotland	1.051	(0.154)	[0.79–1.40]	1.016	(0.127)	[0.80–1.30]	1.236*	(0.129)	[1.01–1.52]
Constant	0.586	(0.281)	[0.23–1.50]	0.131***	(0.058)	[0.06–0.31]	1.202	(0.411)	[0.62–2.35]
N	5396			5651			6018		
Pseudo R^2^	0.09			0.14			0.06		
Log Likelihood	− 2290.0			− 2675.8			− 3630.1		

Data from 1970 British Cohort Study (sweep 1–4, 8–9). In model 1, voter turnout is in reference to the 2015 elections for participants asked prior to October 2017 (n = 4,251), and to the 2017 British general elections for participants asked after October 2017 (n = 4,237). In model 2, voter turnout is in reference to the 2010 elections. In model 3, voter turnout is in reference to the 1997 general election. Standard Error in parenthesis

^+^p < 0.1; *p < 0.05; **p < 0.01; ***p < 0.001

### Propensity Score Matching (PSM)

Finally, we conducted comparisons using PSM with the normative group as the reference group in both models. Matching variables included participants’ sex, social class at birth, family composition, maternal age at first birth, maternal age when left education, and a proxy of the participant’s cognitive ability at age 5. Additional information regarding balance checks and post-matching model quality is provided in the supplementary material (Appendix 2 and eFigures 2–4). Following matching, differences were found between groups and were in the expected direction. At age 30, participants in the elevated-chronic group (Model 2) were less likely to vote (i.e., a mean difference of 20%) as were participants in the moderate-chronic group (i.e., a mean difference of 7%), as compared to the normative group. At age 42, participants in the elevated-chronic group (Model 2) were less likely to vote (i.e., a mean difference of 16%) as were participants in the moderate-chronic group (i.e., a mean difference of 5%), as compared to the normative group. Participants in the elevated-chronic group (Model 2) were less likely to vote at age 46 (i.e., a mean difference of 14%), as were participants in the moderate-chronic group (i.e., a mean difference of 4%), as compared to the normative group. Please see Tables 3, 4, and 5.

**Table 3:** Conduct problems and voter turnout in 1997: Age 30 pre and post matching results

	Pre matching				Post matching			
	T	C	Diff (Sig.)	S.E	T	C	Diff (Sig.)	S.E
Model 1: Group 2								
Voter turnout	0.55	0.65	− 0.10***	0.01	0.55	0.62	− 0.07***	0.02
Model 2: Group 3								
Voter turnout	0.42	0.65	− 0.23***	0.04	0.41	0.61	− 0.20***	0.05

***Significance at the p < 0.001 level, ** at the 0.01 level. T denotes ‘treatment’ (conduct problems) and C denotes ‘control’ (normative group). ‘Diff’ represents the difference in scores between groups. S.E. refers to the standard errors. N for the treatment group in Model 1 (Group 2) was 1782 and 6863 for the control group as 1 participant in the treatment group was off support. N for the treatment group in model 2 (Group 3) was 129 and 6863 for the control group as 1 participant in the treatment group was off support

**Table 4:** Conduct problems and voter turnout in 2010: age 42 pre and post matching results

	Pre matching				Post matching			
	T	C	Diff (Sig.)	S.E	T	C	Diff (Sig.)	S.E
Model 1: Group 2								
Voter turnout	0.67	0.77	− 0.10***	0.01	0.67	0.71	− 0.05***	0.02
Model 2: Group 3								
Voter turnout	0.55	0.77	− 0.22***	0.05	0.54	0.7	− 0.16**	0.07

***Significance at the p < 0.001 level, ** at the 0.01 level. T denotes ‘treatment’ (conduct problems) and C denotes ‘control’ (normative group). ‘Diff’ represents the difference in scores between groups. S.E. refers to the standard errors. N for the treatment group in Model 1 (Group 2) was 1305 and 5278 for the control group. N for the treatment group in model 2 (Group 3) was 70 and 5278 for the control group, as 1 participant in the treatment group was off support

**Table 5:** Conduct problems and voter turnout in 2017/2015: age 46 pre and post matching results

	Pre matching					Post matching		
	T	C	Diff (Sig.)	S.E	T	C	Diff (Sig.)	S.E
Model 1: Group 2								
Voter turnout	0.75	0.83	− 0.08***	0.01	0.75	0.79	− 0.04***	0.01
Model 2: Group 3								
Voter turnout	0.63	0.83	− 0.21***	0.04	0.63	0.77	− 0.14**	0.06

***Significance at the p < 0.001 level, ** at the 0.01 level. T denotes ‘treatment’ (conduct problems) and C denotes ‘control’ (normative group). ‘Diff’ represents the difference in scores between groups. S.E. refers to the standard errors. N for the treatment group in Model 1 (Group 2) was 1299 and 5240 for the control group. N for the treatment group in Model 2 (Group 3) was 80 and 5240 for the control group

## Discussion

The main contribution of this paper is the longitudinal negative association between developmental trajectories of conduct problems (from age 5 to 16) and political participation (at ages 30, 42, and 46). This is the first study to the best of our knowledge that uses a lifespan approach crossing five of the six developmental periods (i.e., from birth to middle age), to examine whether trajectories of conduct problems map onto voter turnout at different periods across adulthood. We use a variety of robust statistical modelling techniques (i.e., multivariable logistic regression, linear probability models, PSM), continually arriving at the same conclusions. Thus, our results shed several important new insights and are discussed in turn.

First, we find a 3-group trajectory model of conduct problems best fits the data. We use a person-centred approach, which is particularly well suited to examining the heterogeneity between groups in the evolution of conduct problems over time. The identified trajectory groups are similar to those previously reported (Maughan et al., 2000), albeit with a slightly smaller prevalence rate in the elevated-chronic group. This is not surprising given the increasing prevalence of conduct problems reported since the childhood data was collected (Collishaw et al., 2004). We find some support for Moffitt’s developmental taxonomy (1996, 2015) with respect to an early onset persistent group (group 3, the elevated-chronic group). However, within the current cohort no support for an adolescent-onset group was found.

Second, we find a negative robust association between trajectories of conduct problems and voter turnout 14, 26, and 30 years later, independent of important covariates such as family socio-demographics, cognitive ability and education level, social class, income, proximal mental health, and political interest. Specifically, at age 30, participants in both the elevated-chronic and moderate-chronic groups were less likely to participate in elections, as compared to the normative group. The adjusted difference in turnout rates for the elevated-chronic and moderate-chronic groups resulted in reduced odds of voting of 52.2% and 24.7%., respectively. This would suggest a possible dose–response association (e.g., as the level of conduct problems increase, the level of voter turnout decreases) for voter turnout at 30 years. At ages 42 and 46, only participants in the elevated-chronic group were found to be less likely to participate in elections in our main multivariable logistic regression models, with the adjusted difference reducing their odds by 52.0% and 45.7%, respectively. Taken together, this would suggest that children with early onset chronic conduct problems, are those most at risk of not voting across adulthood. Considering that participants with conduct problems were less likely to report on turnout and had higher rates of attrition (i.e., only 36.8% of participants in the elevated-chronic group had information on turnout and 48.2% in the moderate-chronic group as compared to 53.9% in the normative group), coupled with the increasing prevalence rate of conduct problems since the 1970s, the magnitude of our findings may be larger than reported here in younger generations. For example, identified trajectory groups (i.e., number of groups and evolution over time) may present differently with younger cohorts given this observed rise in conduct problems, which have been found across gender, class, and family composition (Collishaw et al., 2004). Thus, this study warrants replication using younger cohorts to examine whether trajectory groups identified would be similar to those found in the current cohort, and whether the magnitude of effect would be larger than observed here.

Third, given the importance of early family characteristics and cognitive ability for both conduct problems and political participation, we replicate our findings using a quasi-experimental statistical approach (i.e., PSM). PSM reduces bias between groups by comparing participants who have similar propensities of having conduct problems to those who do not, based on their measured characteristics. Our findings demonstrate a 14–20% difference on adult voter turnout for the elevated-chronic group as compared to the normative group between the ages of 30–46, and a 4–7% difference for the moderate-chronic group between the ages of 30–46. This would suggest a robust negative association across three decades, particularly for those with the most elevated and stable conduct problems. These results are further substantiated when using linear probability models, which also allows for the inclusion of adult socio-demographics, more proximal mental health, and political behaviour given that no restrictions of temporal order are placed when using this type of modelling.

To date the literature on MH and voter turnout has focused almost exclusively on internalising forms of MH such as depression (e.g., Landwehr & Ojeda, 2021; Ojeda, 2015; Ojeda & Slaughter, 2019). This literature has been incredibly important in demonstrating that mental health, in particular depression, is an important antecedent factor for participation in elections. Our results add to this growing field by demonstrating that childhood externalising problems such as conduct problems, which have never previously been examined, also have negative consequences for adult voting behaviour. Moreover, these results hold after controlling for more proximal adult psychiatric morbidity, which includes depression. Given the elapsed time between childhood conduct problems and voter turnout, the effect is arguably sizeable and would suggest the importance of not overlooking earlier periods of development when examining mental health as an antecedent of turnout. Particularly so given that a majority of all adult MH difficulties will have already emerged by adolescence.

The main mechanisms previously suggested for the association between internalising MH difficulties such as depression and turnout include the perceived high cost (i.e., effort required), lower motivation, and reduced cognitive abilities (e.g., Denny & Doyle, 2008; Ojeda, 2015). We propose that some similar mechanisms may be at play in the association between conduct problems and voter turnout, namely cognitive and non-cognitive skills. However, we suggest additional mechanisms (i.e., the repetitive violation of social rules and norms mapping onto a reduced sense of civic duty, poorer social capital, and being less targeted) may also be responsible. It is likely that these suggested mechanisms are intertwined in the case of conduct problems in particular, which warrants a call for closer attention to modelling the interplay of these mechanisms going forward.

Through the modelling approach used in the current study, namely group-based trajectories, we were able to capture both chronicity and severity of conduct problems from early childhood through to adolescence, rather than ‘episodic’ conduct problems at one point in development. Our results indeed demonstrate that both chronicity and severity of conduct problems are factors which are important to consider when examining the association with voter turnout. Arguably, this approach would also be well suited for extending the literature examining internalising forms of MH such as depression and turnout. The examination of both forms of MH difficulties in early development (i.e., via trajectories of externalising *and* internalising difficulties) and its impact on turnout across the lifespan is also needed in future studies given the demonstrated comorbidity between these forms of MH (e.g., Girard, 2021).

Taken together, our results have direct implications regarding another long-term maladaptive outcome for children with conduct problems (i.e., reduced political participation), subsequently affecting the health of democracy. The finding that groups with conduct problems are self-marginalised in terms of political participation, may lead to a lack of inclusiveness for them in the policy decision-making process. This would infer a perpetuating cycle, whereby groups that tend not to participate in elections provide fewer incentives for policy makers to represent their interests. In turn, a lack of policies crucial for these groups to improve their status in society may lead to further apathy in political participation. Such a perpetuating cycle carries with it a high socio-economic burden on society. But if we could instil an early sense of political responsibility (i.e., engagement) in these groups, perhaps they may be more inclined to use positive outlets such as governments to advocate policies in support of their best interests (e.g., social support programmes), reducing long-term overuse of public services (e.g., health, criminal justice system). The whole idea of democratic inclusiveness is to address the problems of marginalised groups through institutional inclusion, and subsequent implementation of policies to improve their socio-economic status within society. Voting would therefore provide an outlet for people with conduct problems to be better represented in society, and for policies of specific interest to them to be implemented/supported. However, a lack of political participation limits the positive impact that democratic inclusion can provide.

Despite several inherent strengths of this study, including the use of a population-based birth cohort, followed longitudinally from birth to 46 years of age, using a repeated measures design, with multiple informants to reduce potential shared method variance, the amount and type of covariates controlled, along with the use of multiple robust statistical techniques, some limitations are noteworthy. First, attrition and item non-response across sweeps resulted in the loss of over half the participants in the initial cohort, with higher attrition amongst participants in the elevated-chronic group, and who were male, with lower cognition, from families with lower parental social class, single parent families, whose mothers were younger at the birth of their first child and when leaving education. Thus, generalizability to the larger population warrants caution. Second, parent-reports alone were used to collect information on conduct problems. Ideally, the use of multiple informants (e.g., teacher-reports) would have strengthened the findings. Third and relatedly, the scaling of the Rutter A2 parent-report was changed at age 10 to a visual analogue scale. While rescaling techniques were used, issues with reliability may still be present. Fourth, PSM is only able to match on observables. Unobservables not accounted for may contribute to the association found. Fifth, the use of self-reports for turnout can introduce bias within the model results given the potential for overreporting of turnout, particularly amongst those with higher levels of political interest and education (e.g., Enamorando & Imai, 2019), the latter of which is associated with lower levels of conduct problems. Thus, replication with objective registry data on voter turnout is suggested. Finally, an early general election was called during data collection at age 46, resulting in this turnout question referencing the 2015 election only for some participants and both the 2015 and 2017 election for others. However, to ensure fidelity of our findings, we also conduct sensitivity analysis using voter turnout in 2015 alone and examine turnout out at ages 30 and 42.

Considering these limitations, this study contributes important new insights to the literature examining the critical age old question of ‘who abstains from voting’. Namely, we identified that childhood MH difficulties such as conduct problems contribute to lower levels of voter turnout in adulthood, lending towards marginalisation through institutional exclusion in the democratic process. This novel area of research is one in which requires greater attention given the high personal and societal costs of having marginalised groups within democracies (e.g., further isolation from society; use of alternative behaviours such as crime to achieve needs). In addition to our results shedding light on the importance of childhood conduct problems in particular, our results further highlight the importance of examining childhood characteristics, rather than focusing uniquely on more proximal antecedent risk and protective factors for adult turnout. Indeed, adolescence is becoming a developmental period of high interest within the turnout literature (e.g., Holbein & Hillygus, 2020; Okolikj & Hooghe, 2022), however we may be able to learn even more by examining factors that present in childhood. Future research should also look at different aspects of early political engagement such as volunteering, protests, online activism, and group activities to better understand how conduct problems in childhood and adolescence may be implicated more holistically for adult civic behaviours. Additionally, future studies ought to examine the specific impact of antisocial personality disorder in adulthood and its association with voter turnout given the current findings of an association with childhood conduct problems. Moreover, future studies would do well to build upon the current work by also examining trajectories of adult voting behaviour across early, mid, and late adulthood for those with the most elevated and chronic levels of childhood conduct problems, to better understand continuity of non-engagement in voter turnout. Given the multifactorial nature of both mental health conditions such as conduct problems and political behaviours such as voting, a call towards greater interdisciplinary efforts to advance knowledge across fields is warranted.

## Supplementary Information

Below is the link to the electronic supplementary material.

Supplementary file1 (DOCX 486 KB)

## Data Availability

Dr. Girard and Dr. Okolikj had full access to all the data in the study and take responsibility for the integrity of the data and the accuracy of the data analysis.

## Footnotes

1 For justification of control variables see Appendix 1 in the supplementary material.

2 The replication code for all data analyses conducted in this study can be found at: https://doi.org/10.7910/DVN/S6UUBF. The proprietary data used in this study are under Special Licence agreement and thus cannot be shared directly by the authors. Access can however be gained directly via application to the UK dataservice under their Special End User Licence which can be found here: http://discover.ukdataservice.ac.uk/series/?sn=200001.

3 GBTM is a data driven and theory-based technique that identifies latent groups of individuals who are following the same developmental pattern of growth (i.e., trajectories) over time. Both the level of intensity of conduct problems, along with the growth pattern (i.e., increasing, decreasing, quadratic, stable) are simultaneously modelled within GBTM. Whilst linear trajectories were observed, the pattern in this cohort was that of decreasing rather than increasing. The likelihood of correct classification of individuals to the latent groups are assessed using two model fit indices, namely, the average posterior probability of group membership and the odds of correct classification.
