# Supplementary material for: Trajectories of Mental Health Problems in Childhood and Adult Voting Behaviour: Evidence from the 1970s British Cohort Study
Source: Polit Behav. 2023 Jan 21:1–24. Online ahead of print. doi: 10.1007/s11109-022-09852-9 (PMC9862212; doi:10.1007/s11109-022-09852-9)
Supplement: Supplementary file 1 — Supplementary file1 (DOCX 486 KB) [file 11109_2022_9852_MOESM1_ESM.docx]

Table of Contents

[Supplementary material 2](#_Toc121311897)

[Appendix 1. Justification of Control variables 2](#_Toc121311898)

[eTable 1. Multivariable logistic regression model: Conduct problems and voter turnout in the 2015 British General Elections. 3](#_Toc121311899)

[eTable 2. Conduct problems and voter turnout 2015: Pre and post matching results 4](#_Toc121311900)

[eFigure 1. Rescaling of items at age 10: From a visual analog scale to categorical. 5](#_Toc121311901)

[eTable 3. Bayesian information criteria (BIC) for assessment of trajectory model fit. 7](#_Toc121311902)

[eTable 4. Model fit criterion: Conduct problem group-based trajectories. 7](#_Toc121311903)

[eTable 5. Multivariable logistic regression model: Conduct problems and voter turnout in the 2015/2017, 2010, and 1997 British General Elections using variables that precede Conduct problems. 8](#_Toc121311904)

[eTable 6. Demographic and political characteristics by trajectory group membership at age 30 9](#_Toc121311905)

[eTable 7. Demographic and political characteristics by trajectory group membership at age 42 10](#_Toc121311906)

[eTable 8. Demographic and political characteristics by trajectory group membership at age 46 11](#_Toc121311907)

[eTable 9. Multivariable linear probability regression model with robust standard errors: Conduct problems and voter turnout in the British General Elections. 12](#_Toc121311908)

[Appendix 2. PSM Balance Checks and Model Quality 14](#_Toc121311909)

[eFigure 2. Standardised differences across covariates: Pre and post-matching at age 30 14](#_Toc121311910)

[eFigure 3. Standardised differences across covariates: Pre and post-matching at age 42 15](#_Toc121311911)

[eFigure 4. Standardised differences across covariates: Pre and post-matching at age 46 15](#_Toc121311912)

# Supplementary material

# Appendix 1. Justification of Control variables

Family demographic characteristics have been shown to have a direct influence on children’s political behaviour (Jennings and Niemi 1968; Jennings and Niemi 2014). This line of research suggests that transfer of political orientation is robust across generations (Jennings, Stoker and Bowers 2009), inherent in the family composition (Wolfinger and Wolfinger 2008; Sandell and Plutzer 2005), and influenced by parental social class status (Parry, Moyser and Day 1992). We include several covariates to capture and account for socio-demographic characteristics of the participant’s parent(s) and the family circumstances in which participants were raised. This includes family composition, maternal age at first birth, maternal age when left education, and social class of parents. We also include participant characteristics (i.e., sex, cognitive ability, level of education, SES/income) given 1) the ongoing debate about sex differences in electoral participation and political interest (Verba, Nie and Kim 1978; Kostelka, Blais and Gidengil 2019), and 2) the large body of literature identifying the importance of cognitive ability, education level, and SES for participation (e.g., Deary, Batty and Gale, 2008; Smets, 2013; Brady, Verba and Schlozman 1995). Further, we include two strong political predictors of electoral participation, namely political interest (Denny and Doyle 2008) and union membership (Anzia 2011). Interest in politics results in a higher level of knowledge about the political system and the competing political parties, with better-informed people having a higher propensity to vote (Lassen 2005). Unions have a long history of influencing their members to participate in elections, which is particularly evident in earlier cohorts (Flavin and Radcliff 2011; Leighley and Nagler 2007). Additionally, we control for more proximal mental health (i.e., psychiatric morbidity) in adulthood given the emerging literture on adult mental health and voter turnout (e.g., Denny and Doyle, 2008; Landwehr & Ojeda, 2021). Finally, we control for geographical differences across regions in Great Britain, distinguishing between participants born in England, Scotland and Wales.

In the same vein, the developmental psychology and psychiatry literature suggests that there are certain family, maternal and child characteristics that are common amongst children with conduct problems and these factors have been identified as increasing the risk of membership in groups with elevated problems. For example, family composition (i.e., single parents) (Loeber and Stouthamer-Loeber 1986) and lower social class (Murray, et al., 2013), have been found to increase the risk of conduct problems in childhood. Moreover, poorer cognitive ability (Fairchild et al. 2019; Moffit, 1993), lower maternal education (Tremblay, et. al. 2004) and age (Shaw, Lacourse and Nagin 2005) have conferred increased risk. Finally, boys are at substantially higher risk as compared to girls (Moffitt, Caspi, Dickson, Silva and Stanton 1996; Girard, et al., 2019).

# eTable 1. Multivariable logistic regression model: Conduct problems and voter turnout in the 2015 British General Elections.

|  | Turnout at 46 | | |
| --- | --- | --- | --- |
| Variable | OR | *SE* | 95% CI |
| *Conduct Problems (ref. Normative)* |  |  |  |
| Moderate-chronic | 0.79** | (0.071) | [0.66-0.94] |
| Elevated-chronic | 0.468** | (0.135) | [0.27-0.82] |
| *Sex (ref. Male)* |  |  |  |
| Female | 1.490*** | (0.113) | [1.28-1.73] |
| *Education (ref. Less than high-school)* |  |  |  |
| More than high-school | 1.87*** | (0.168) | [1.57-2.23] |
| Not stated | 1.208 | (0.213) | [0.86-1.71] |
| *Copy Design (standardized)* | 1.042 | (0.042) | [0.96-1.13] |
| *Maternal Age at First Birth* | 1.031** | (0.011) | [1.01-1.05] |
| *Social Class at Birth*  *(ref. Managerial/Professional)* |  |  |  |
| Non-manual/manual | 0.843 | (0.094) | [0.68-1.05] |
| Unskilled/partly-skilled | 0.715** | (0.095) | [0.55-0.93] |
| Not working/Other | 0.356* | (0.187) | [0.13-1.00] |
| *Married (ref.)* |  |  |  |
| Single (includes: widowed, divorced, separated) | 0.943 | (0.162) | [0.67-1.32] |
| *Maternal Age when Left Education* | 1.051* | (0.027) | [1.0-1.11] |
| *Trade Union (ref. not a member)* |  |  |  |
| Member | 1.291* | (0.150) | [1.03-1.62] |
| Not stated | 1.099 | (0.292) | [0.65-1.85] |
| *Political Interest (ref. not interested)* |  |  |  |
| Interested | 3.640*** | (0.358) | [3.00-4.41] |
| Not stated | 0.940 | (0.265) | [0.54-1.63] |
| *Psychiatric Morbidity (high)* | 0.752** | (0.081) | [0.61-0.93] |
| *Socio-economic-status* |  |  |  |
| Total Income Household | 1.000*** | (0.000) | [1.00-1.00] |
| *Region of Birth (ref. England)* |  |  |  |
| Wales | 1.160 | (0.192) | [0.84-1.60] |
| Scotland | 1.214 | (0.181) | [0.91-1.63] |
| Constant | 0.51 | (0.244) | [0.20-1.30] |
| *N* | 5,389 | | |
| *Pseudo R^2^* | 0.106 | | |
| *Log Likelihood* | -2325.7 | | |

Note: Data from 1970 British Cohort Study (sweep 1-4, 8-9). The dependent variable is in reference to vote turnout for the 2015 British general elections only and does not consider electoral participation in the 2017 general election.

# eTable 2. Conduct problems and voter turnout 2015: Pre and post matching results

|  | Pre Matching | | | | Post Matching | | | |
| --- | --- | --- | --- | --- | --- | --- | --- | --- |
|  | T | C | Diff (Sig.) | S.E | T | C | Diff (Sig.) | S.E |
| Model 1:  Group 2 |  |  |  |  |  |  |  |  |
| Voter turnout | 0.73 | 0.82 | -0.09*** | 0.01 | 0.73 | 0.79 | -0.06*** | 0.02 |
| Model 2: Group 3 |  |  |  |  |  |  |  |  |
| Voter turnout | 0.6 | 0.82 | -0.22*** | 0.04 | 0.6 | 0.75 | -0.15** | 0.07 |

Note: *** denotes significance at the p <0.001 level, ** at the 0.01 level. T denotes ‘treatment’ (conduct problems) and C denotes ‘control’ (normative group). ‘Diff’ represents the difference in scores between groups. S.E. refers to the standard errors. N for the treatment group in Model 1 (Group 2) was 1,298 and 5,232 for the control group, as 1 participant in the treatment group was off support. N for the treatment group in model 2 (Group 3) was 80 and 5,232 for the control group.

# eFigure 1. Rescaling of items at age 10: From a visual analog scale to categorical.

| Original distribution | Re-scaling to three categories |
| --- | --- |
| 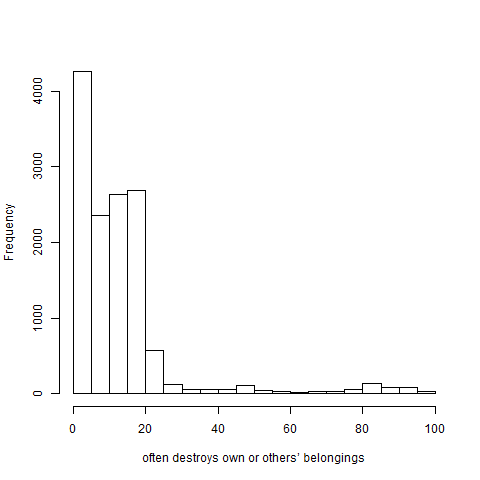 | 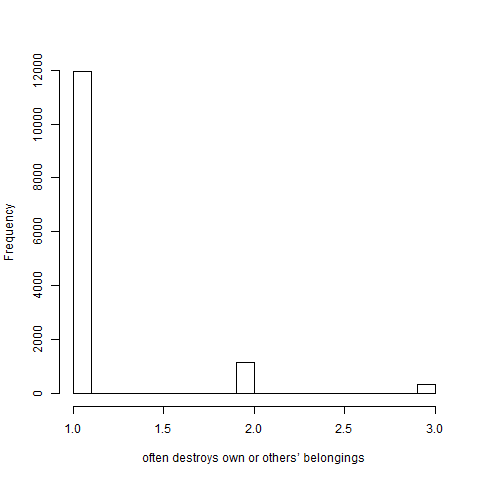 |
| 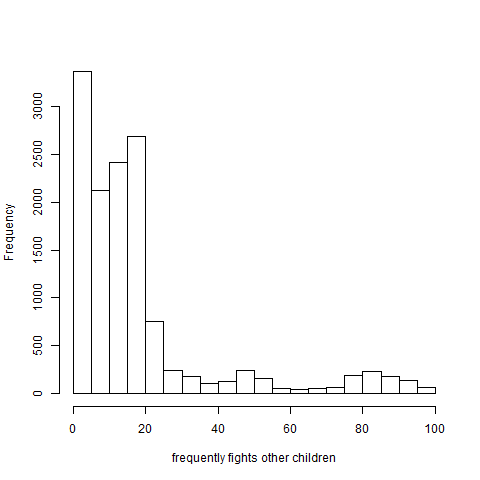 | 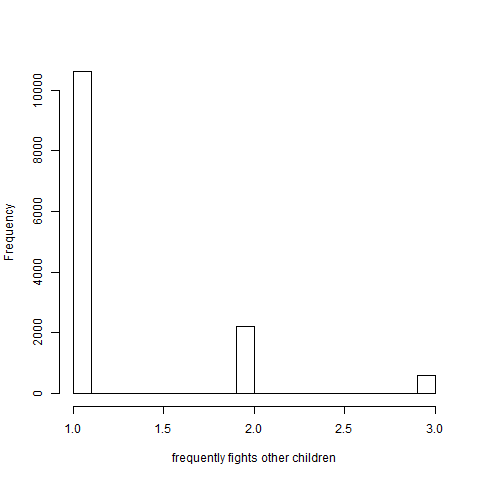 |
| 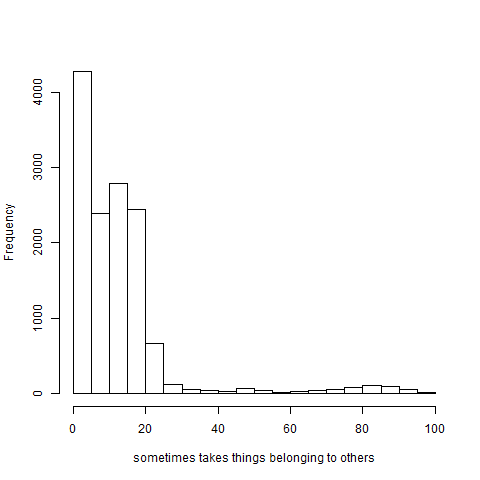 | 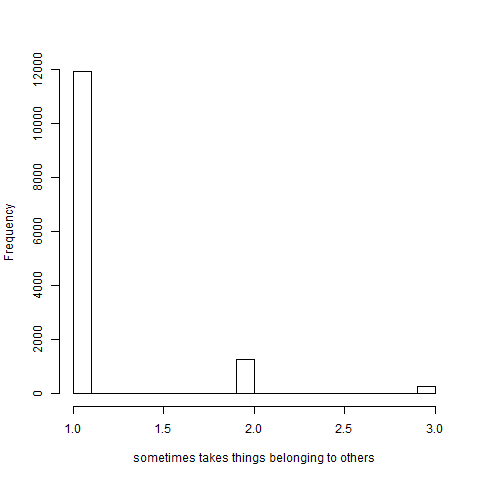 |
| 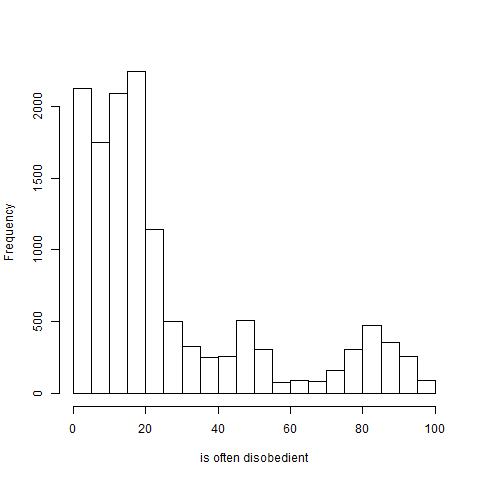 | 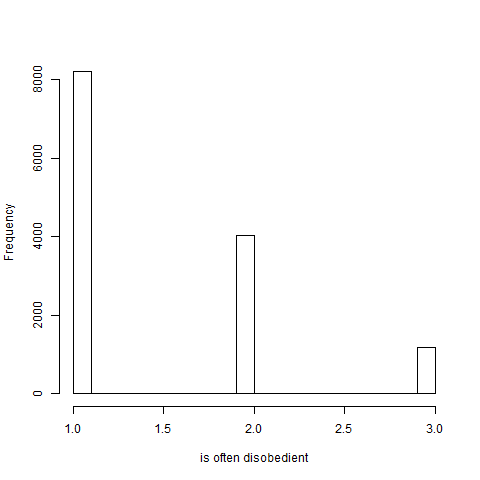 |
| 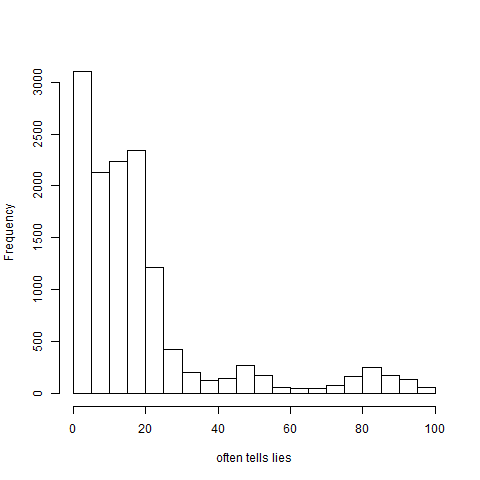 | 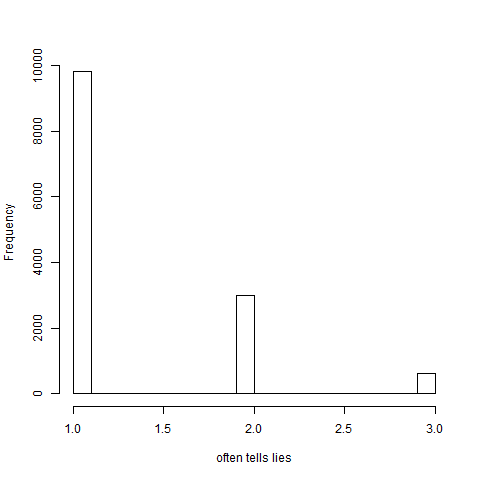 |
| 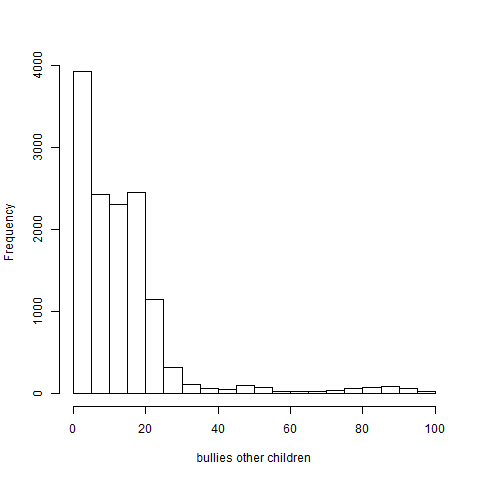 | 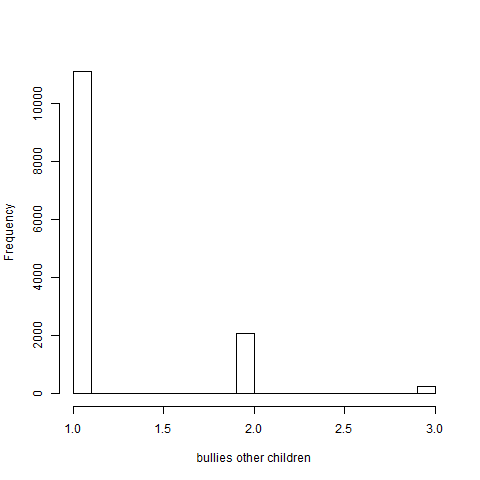 |

Note: Rescaling of the visual analog scale for individual behavioural items from 0 to 100 into three categories was done for age 10 data, in order to be comparable to age 5 and age 16 data, when behavioural items were rated on a 3-point Likert scale. Three cut-off points were used: 0 to 20 coded as 1; 21 to 80 coded as 2; and 81 to 100 coded as 3. After the rescaling, we compared the age 10 categorical variable, with those distributions from age 5 and 16. Theoretically, it is expected that a large proportion of children with early moderate and elevated conduct problems will decline over time, so cut-off points were selected following this theoretical expectation. We found that the average level for all 6 items on conduct problems following this theoretical expectation was situated within the above cut-off points. The categorization of the variables were done prior to trajectory group modeling.

# eTable 3. Bayesian information criteria (BIC) for assessment of trajectory model fit.

| Conduct Problems | BIC (N=34496) | BIC (N=15554) | AIC |
| --- | --- | --- | --- |
| 2 group | -22993.98 | -22990.80 | -22960.19 |
| 3 group | -22479.21 | -22474.43 | -22428.52 |
| 4 group | -22446.02 | -22439.64 | -22378.43 |
| 5 group | -22326.48 | -22318.52 | -22242.00 |
| 6 group | -22347.38 | -22337.82 | -22246.00 |

Note: A larger (more positive) BIC and AIC is indicative of better model fit. The smaller N BIC represents the actual sample size used in the trajectories, the larger N BIC represents the total number of assessments used within the estimation of the model across time and participants. The two presented BIC scores bracket the theoretically correct BIC score (Nagin, 2005).

# eTable 4. Model fit criterion: Conduct problem group-based trajectories.

| Trajectory Group | *n* | Average Posterior Probability of Group Membership | Odds of Correct Classification |
| --- | --- | --- | --- |
| 1 | 11,871 | 86.5 | 2 |
| 2 | 3,433 | 72.8 | 9.5 |
| 3 | 250 | 77.7 | 213 |

Note: Membership probability greater than 70 and OCC greater than 5 represent good model fit.

# eTable 5. Multivariable logistic regression model: Conduct problems and voter turnout in the 2015/2017, 2010, and 1997 British General Elections using variables that precede Conduct problems.

|  | Turnout at 46 | | | | Turnout at 42 | | | | | Turnout at 30 | | |
| --- | --- | --- | --- | --- | --- | --- | --- | --- | --- | --- | --- | --- |
| Variable | OR | *SE* | | 95% CI | OR | *SE* | | | 95% CI | OR | *SE* | 95% CI |
| *Conduct Problems (ref. Normative)* |  | | | |  | | | | |  |  |  |
| Moderate-chronic | 0.750*** | | (0.058) | [0.65-0.87] | 0.752*** | | (0.053) | | [0.66-0.86] | 0.714*** | (0.040) | [0.64-0.80] |
| Elevated-chronic | 0.523** | | (0.125) | [0.33-0.83] | 0.564* | | (0.139) | | [0.35-0.91] | 0.480*** | (0.088) | [0.34-0.69] |
| *Sex (ref. Male)* |  | | |  |  | | | |  |  |  |  |
| Female | 1.371*** | | (0.089) | [1.21-1.56] | 1.060 | | (0.062) | | [0.95-1.19] | 1.210*** | (0.088) | [1.11-1.32] |
| *Copy Design (standardized)* | 1.159*** | | (0.040) | [1.08-1.24] | 1.165*** | | (0.036) | | [1.10-1.24] | 1.071* | (0.025) | [1.02-1.05] |
| *Maternal Age at First Birth* | 1.057*** | | (0.010) | [1.04-1.08] | 1.065*** | | (0.009) | [1.05-1.08] | | 1.036*** | (0.006) | [1.02-1.05] |
| *Social Class at Birth*  *(ref. Managerial/Professional)* |  | | | |  | | | | |  |  |  |
| Non-manual/manual | 0.754** | | (0.073) | [0.62-0.91] | 0.680*** | | (0.059) | | [0.57-0.81] | 0.911 | (0.058) | [0.80-1.03] |
| Unskilled/partly-skilled | 0.634*** | | (0.073) | [0.51-0.80] | 0.616*** | | (0.064) | | [0.50-0.75] | 0.850* | (0.067) | [0.73-0.99] |
| Not working/Other | 0.465 | | (0.212) | [0.19-1.14] | 0.441 | | (0.190) | | [0.19-1.03] | 0.711 | (0.238) | [0.37-1.37] |
| *Married (ref.)* |  | | | |  | | | | |  |  |  |
| Single (includes: widowed, divorced, separated) | 0.880 | | (0.122) | [0.67-1.15] | 0.863 | | (0.108) | | [0.68-1.10] | 0.999 | (0.107) | [0.81-1.23] |
| *Maternal Age when Left Education* | 1.073*** | | (0.022) | [1.03-1.12] | 1.104*** | | (0.022) | | [1.06-1.15] | 1.021 | (0.014) | [0.99-1.05] |
| *Region of Birth (ref. England)* |  | | | |  | | | | |  |  |  |
| Wales | 1.125 | (0.162) | | [0.85-1.49] | 1.442* | | (0.197) | | [1.10-1.89] | 1.422*** | (0.137) | [1.18-1.72] |
| Scotland | 1.313* | (0.167) | | [1.02-1.69] | 1.151 | | (0.123) | | [0.93-1.42] | 1.271* | (0.103) | [1.09-1.49] |
| Constant | 0.501 | (0.199) | | [0.23-1.09] | 0.218*** | | (0.081) | | [0.11-0.45] | 0.566* | (0.149) | [1.09-1.49] |
| *N* | 6,612 | | | | 6,651 | | | | | 8,772 | | |
| *Pseudo R^2^* | 0.04 | | | | 0.04 | | | | | 0.02 | | |
| *Log Likelihood* | -3073.7 | | | | -3624.2 | | | | | -5704.4 | | |

Note: Data from 1970 British Cohort Study (sweep 1-4, 6, 8-9). *p<0.05; **p<0.01; ***p<0.001.

# eTable 6. Demographic and political characteristics by trajectory group membership at age 30

|  | Conduct Problems | | |  |
| --- | --- | --- | --- | --- |
|  | Normative | Moderate-chronic | Elevated-chronic | p-value |
| *Sex of Participant* |  |  |  | <0.001 |
| Male | 2017 | 597 | 45 |  |
|  | (41.60) | (53.78) | (72.58) |  |
| Female | 2832 | 513 | 17 |  |
|  | (58.40) | (46.22) | (27.42) |  |
| *Parental Social Class* |  |  |  | <0.001 |
| Professional/managerial | 1064 | 152 | 4 |  |
|  | (21.94) | (13.69) | (6.45) |  |
| Non-manual/manual | 2944 | 682 | 34 |  |
|  | (60.71) | (61.44) | (54.84) |  |
| Unskilled/partly skilled | 821 | 271 | 20 |  |
|  | (16.93) | (24.41) | (32.26) |  |
| Not working/Other | 20 | 5 | 4 |  |
|  | (0.41) | (0.45) | (6.45) |  |
| *Family Structure* |  |  |  | <0.001 |
| Married | 4690 | 1044 | 58 |  |
|  | (96.72) | (94.05) | (93.55) |  |
| Single/divorced/widowed | 159 | 66 | 4 |  |
|  | (3.28) | (5.95) | (6.45) |  |
| *Education* |  |  |  | <0.001 |
| Low | 2845 | 785 | 39 |  |
|  | (58.67) | (70.72) | (62.90) |  |
| High | 1780 | 220 | 7 |  |
|  | (36.71) | (19.82) | (11.29) |  |
| Non respondents | 224 | 105 | 16 |  |
|  | (4.62) | (9.46) | (25.81) |  |
| *Trade Union Membership* |  |  |  | <0.01 |
| Not a member | 3711 | 895 | 54 |  |
|  | (76.53) | (80.63) | (87.10) |  |
| Member | 1138 | 215 | 8 |  |
|  | (23.47) | (19.37) | (12.90) |  |
| *Political Interest* |  |  |  | =0.229 |
| Not interested | 1657 | 350 | 22 |  |
|  | (34.19) | (31.53) | (35.48) |  |
| Interested | 3189 | 760 | 40 |  |
|  | (65.81) | (68.47) | (64.52) |  |
| Mean (SD) |  |  |  |  |
| *Maternal Age at First Birth* | 22.89  (4.01) | 21.80  (3.79) | 20.19  (3.54) | <0.001 |
| *Maternal Age when Left Education* | 15.82  (1.90) | 15.60  (1.75) | 15.32  (1.34) | <0.001 |
| *Copy Design Cognition* | 0.20  (0.97) | -0.11  (1.00) | -0.54  (0.95) | <0.001 |
| *Psychiatric Morbidity* | 0.10  (0.31) | 0.16  (0.37) | 0.21  (0.41) | <0.001 |
| *Income Scale* | 1.93  (0.90) | 2.14  (1.01) | 2.36  (1.16) | <0.01 |

Note: Percentage by conduct problems for categorical variables in parenthesis.

# eTable 7. Demographic and political characteristics by trajectory group membership at age 42

|  | Conduct Problems | | |  |
| --- | --- | --- | --- | --- |
|  | Normative | Moderate-chronic | Elevated-chronic | p-value |
| *Sex of Participant* |  |  |  | <0.001 |
| Male | 2000 | 554 | 43 |  |
|  | (43.92) | (53.22) | (76.79) |  |
| Female | 2554 | 487 | 13 |  |
|  | (56.08) | (46.78) | (23.21) |  |
| *Parental Social Class* |  |  |  | <0.001 |
| Professional/managerial | 1026 | 147 | 5 |  |
|  | (22.53) | (14.12) | (8.93) |  |
| Non-manual/manual | 2720 | 621 | 30 |  |
|  | (59.73) | (59.65) | (53.57) |  |
| Unskilled/partly skilled | 794 | 270 | 20 |  |
|  | (17.44) | (25.94) | (35.71) |  |
| Not working/Other | 14 | 3 | 1 |  |
|  | (0.31) | (0.29) | (1.79) |  |
| *Family Structure* |  |  |  | <0.001 |
| Married | 4378 | 964 | 52 |  |
|  | (96.14) | (92.60) | (92.86) |  |
| Single/divorced/widowed | 176 | 77 | 4 |  |
|  | (3.87) | (7.40) | (7.14) |  |
| *Education* |  |  |  | <0.001 |
| Low | 2458 | 751 | 45 |  |
|  | (53.98) | (72.14) | (80.36) |  |
| High | 2096 | 290 | 11 |  |
|  | (46.02) | (27.86) | (19.64) |  |
| *Trade Union Membership* |  |  |  | =0.731 |
| Not a member | 3662 | 838 | 48 |  |
|  | (80.41) | (80.50) | (85.71) |  |
| Member | 789 | 177 | 8 |  |
|  | (17.33) | (17.00) | (14.29) |  |
| Not stated | 103 | 26 | 0 |  |
|  | (2.26) | (2.50) | (0.00) |  |
| *Political Interest* |  |  |  | <0.01 |
| Not interested | 2519 | 622 | 35 |  |
|  | (55.31) | (59.75) | (62.50) |  |
| Interested | 2026 | 411 | 21 |  |
|  | (44.49) | (39.48) | (37.50) |  |
| Not stated | 9 | 8 | 0 |  |
|  | (0.20) | (0.77) | (0.00) |  |
| Mean (SD) |  |  |  |  |
| *Maternal Age at First Birth* | 22.91  (4.01) | 21.76  (3.89) | 12.91  (2.59) | <0.001 |
| *Maternal Age when Left Education* | 15.83  (1.83) | 15.52  (1.74) | 15.20  (1.27) | <0.001 |
| *Copy Design Cognition* | 0.20  (0.96) | -0.12  (0.98) | -0.58  (0.92) | <0.001 |
| *Psychiatric Morbidity* | 0.10  (0.30) | 0.15  (0.36) | 0.21  (0.41) | <0.001 |
| *Social Class Scale* | 2.84  (0.96) | 2.55  (0.90) | 2.45  (0.91) | <0.01 |

Note: Percentage by conduct problems for categorical variables in parenthesis.

# eTable 8. Demographic and political characteristics by trajectory group membership at age 46

|  | Conduct Problems | | |  |
| --- | --- | --- | --- | --- |
|  | Normative | Moderate-chronic | Elevated-chronic | p-value |
| *Sex of Participant* |  |  |  | <0.001 |
| Male | 1967 | 539 | 42 |  |
|  | (45.33) | (53.90) | (73.68) |  |
| Female | 2372 | 461 | 15 |  |
|  | (54.67) | (46.10) | (26.32) |  |
| *Parental Social Class* |  |  |  | <0.001 |
| Professional/managerial | 1002 | 143 | 4 |  |
|  | (23.09) | (14.30) | (7.02) |  |
| Non-manual/manual | 2599 | 618 | 35 |  |
|  | (59.90) | (61.80) | (61.40) |  |
| Unskilled/partly skilled | 723 | 237 | 16 |  |
|  | (16.66) | (23.70) | (28.07) |  |
| Not working/Other | 15 | 2 | 2 |  |
|  | (0.35) | (0.00) | (0.04) |  |
| *Family Structure* |  |  |  | <0.001 |
| Married | 4183 | 927 | 51 |  |
|  | (96.41) | (92.70) | (89.47) |  |
| Single/divorced/widowed | 156 | 73 | 6 |  |
|  | (3.59) | (7.30) | (10.53) |  |
| *Education* |  |  |  | <0.001 |
| Low | 2146 | 668 | 41 |  |
|  | (49.46) | (66.80) | (71.93) |  |
| High | 1970 | 273 | 12 |  |
|  | (45.40) | (27.30) | (21.05) |  |
| Non respondents | 223 | 59 | 4 |  |
|  | (5.14) | (5.90) | (7.02) |  |
| *Trade Union Membership* |  |  |  | <0.001 |
| Not a member | 3052 | 661 | 38 |  |
|  | (70.34) | (66.10) | (66.67) |  |
| Member | 668 | 153 | 4 |  |
|  | (15.40) | (15.30) | (7.02) |  |
| Not stated | 619 | 186 | 15 |  |
|  | (14.27) | (18.60) | (26.32) |  |
| *Political Interest* |  |  |  | <0.001 |
| Not interested | 2051 | 499 | 28 |  |
|  | (47.27) | (49.90) | (49.12) |  |
| Interested | 1744 | 332 | 14 |  |
|  | (40.19) | (33.20) | (24.56) |  |
| Not stated | 544 | 169 | 15 |  |
|  | (12.54) | (16.90) | (26.32) |  |
| Mean (SD) |  |  |  |  |
| *Maternal Age at First Birth* | 22.92  (4.02) | 21.83  (3.85) | 20.14  (3.36) | <0.001 |
| *Maternal Age when Left Education* | 15.87  (1.90) | 15.61  (1.56) | 15.25  (1.20) | <0.001 |
| *Copy Design Cognition* | 0.021  (0.97) | -0.09  (0.98) | -0.58  (0.96) | <0.001 |
| *Psychiatric Morbidity* | 0.10  (0.30) | 0.14  (0.35) | 0.25  (0.43) | <0.001 |
| *Income* | 24,271.82  (66,279.13) | 17,580.33  (35,147.54) | 16,155.18  (39,719.23) | <0.01 |

Note: Percentage by conduct problems for categorical variables in parenthesis.

# eTable 9. Multivariable linear probability regression model with robust standard errors: Conduct problems and voter turnout in the British General Elections.

|  | Turnout at 46 | Turnout at 42 | Turnout at 30 | Turnout at 46 (2015) |
| --- | --- | --- | --- | --- |
| Variable | *Model 1* | *Model 2* | *Model 3* | *Model 4* |
| *Conduct Problems (ref. Normative)* |  |  |  |  |
| Moderate-chronic | -0.028+  (0.014) | -0.031*  (0.015) | -0.063***  (0.016) | -0.041**  (0.015) |
| Elevated-chronic | -0.135*  (0.063) | -0.159*  (0.064) | -0.169**  (0.063) | -0.170**  (0.064) |
| *Sex (ref. Male)* |  |  |  |  |
| Female | 0.060***  (0.010) | 0.043***  (0.011) | 0.057***  (0.012) | 0.054***  (0.011) |
| *Education (ref. Less than high-school)* |  |  |  |  |
| More than high-school | 0.083***  (0.011) | 0.077***  (0.012) | 0.020  (0.014) | 0.079***  (0.011) |
| Not stated | 0.032  (0.033) |  | -0.088**  (0.027) | 0.023  (0.034) |
| *Copy Design (standardized)* | 0.007  (0.006) | 0.007  (0.006) | -0.004  (0.006) | 0.008  (0.006) |
| *Maternal Age at First Birth* | 0.003**  (0.001) | 0.006***  (0.001) | 0.003  (0.002) | 0.004**  (0.001) |
| *Social Class at Birth*  *(ref. Managerial/Professional)* |  |  |  |  |
| Non-manual/manual | -0.005  (0.012) | -0.010  (0.013) | 0.001  (0.016) | -0.015  (0.012) |
| Unskilled/partly-skilled | -0.044**  (0.018) | -0.022  (0.018) | -0.027  (0.021) | -0.044*  (0.018) |
| Not working/Other | -0.100  (0.107) | -0.135  (0.111) | -0.137  (0.093) | -0.183  (0.114) |
| *Married (ref.)* |  |  |  |  |
| Single (includes: widowed, divorced, separated) | -0.016  (0.026) | 0.001  (0.028) | 0.001  (0.032) | -0.008  (0.028) |
| *Maternal Age when Left Education* | 0.004  (0.003) | 0.005*  (0.003) | -0.002  (0.003) | 0.004  (0.003) |
| *Trade Union (ref. not a member)* |  |  |  |  |
| Member | 0.016  (0.013) | 0.062***  (0.013) | 0.068***  (0.014) | 0.027*  (0.013) |
| Not stated | -0.001  (0.036) | 0.107**  (0.032) |  | 0.019  (0.036) |
| *Political Interest (ref. not interested)* |  |  |  |  |
| Interested | 0.126***  (0.011) | 0.227***  (0.011) | 0.211***  (0.012) | 0.154***  (0.011) |
| Not stated | -0.008  (0.040) | 0.196*  (0.093) | -0.553***  (0.014) | -0.016  (0.040) |
| *Psychiatric Morbidity (high)* | -0.039*  (0.018) | -0.027  (0.018) | -0.043*  (0.020) | -0.048**  (0.018) |
| *Socio-economic-status* |  |  |  |  |
| Total Income Household | 0.000**  (0.000) |  |  | 0.000***  (0.000) |
| Social Class |  | 0.025***  (0.006) |  |  |
| Current Financial Situation (reversed) |  |  | -0.017*  (0.007) |  |
| *Region of Birth (ref. England)* |  |  |  |  |
| Wales | 0.021  (0.021) | 0.058*  (0.022) | 0.076*  (0.024) | 0.018  (0.012) |
| Scotland | 0.010  (0.018) | 0.003  (0.020) | 0.045*  (0.021) | 0.029  (0.018) |
| Constant | 0.578***  (0.052) | 0.310***  (0.055) | 0.557***  (0.068) | 0.553***  (0.055) |
| *N* | 5,396 | 5,651 | 6,021 | 5,389 |
| *R squared* | 0.07 | 0.13 | 0.07 | 0.09 |

Note: Data from 1970 British Cohort Study (sweep 1-4, 8-9). In model 1, voter turnout is in reference to the 2015 elections for participants asked prior to October 2017 (n=4,251), and to the 2017 British general elections for participants asked after October 2017 (n=4,237). In model 2, voter turnout is in reference to the 2010 British general elections. In model 3, voter turnout is in reference to the 2015 British general elections. Robust Standard Error in parenthesis. ^+^p<0.1; *p<0.05; **p<0.01; ***p<0.001.

# Appendix 2. PSM Balance Checks and Model Quality

Results of the matching revealed that all participants fell within the area of common support, with the exception of 1 participant in the elevated-chronic group at age 42, 1 participant in the moderate-chronic group at age 30 and 1 participant in the elevated-chronic group at age 30. Balance checks were conducted on individual confounders and the overall model. Remaining bias on individual factors in Model 1 and Model 2 at age 30 ranged between -0.8 and 10.6% and -0.5 and -12.8%, respectively, with the overall remaining mean bias for Model 1 being 3.1% and 5.4% and for Model 2 (eFigure 2). Remaining bias on individual factors in Model 1 and Model 2 at age 42 ranged between 1.4 and 10.2% and 0 and -10.8%, respectively, with the overall remaining mean bias for Model 1 being 4.3% and 4.8% and for Model 2 (eFigure 3). At age 46, remaining bias on individual factors in Model 1 and Model 2 ranged between -0.0 and 5.8% and -0.8 and -17.1%, respectively, with the overall remaining mean bias for Model 1 being 2.2% and 5.5% and for Model 2 (eFigure 4). Remaining bias <20% is indicative of good matching (Rosenbaum and Rubin 1985), thus we concluded successful matching.

# eFigure 2. Standardised differences across covariates: Pre and post-matching at age 30

**
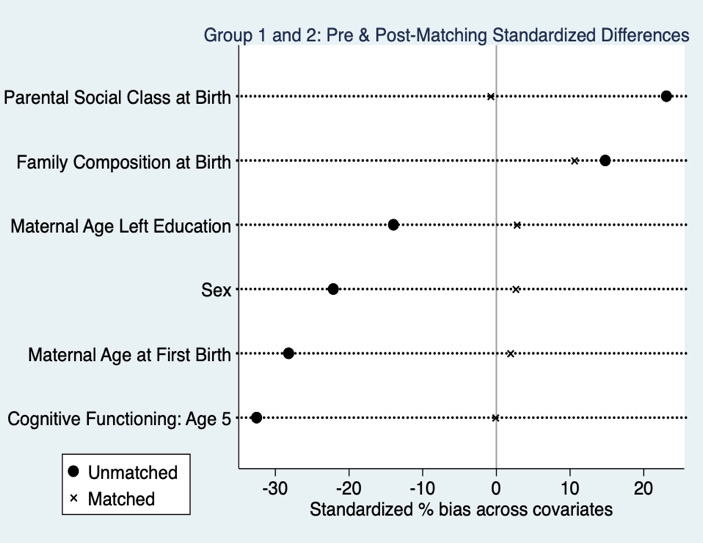

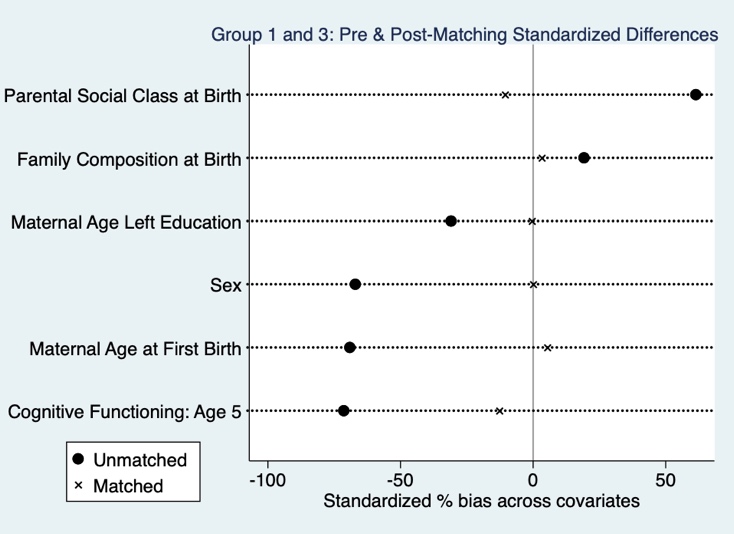
**

Note: N for the treatment group in Model 1 (Group 2) was 1,782 and 6,863 for the control group, as 1 participant in the treatment group was off support. N for the treatment group in model 2 (Group 3) was 129 and 6,863 for the control group, as 1 participant in the treatment group was off support.

# eFigure 3. Standardised differences across covariates: Pre and post-matching at age 42

| 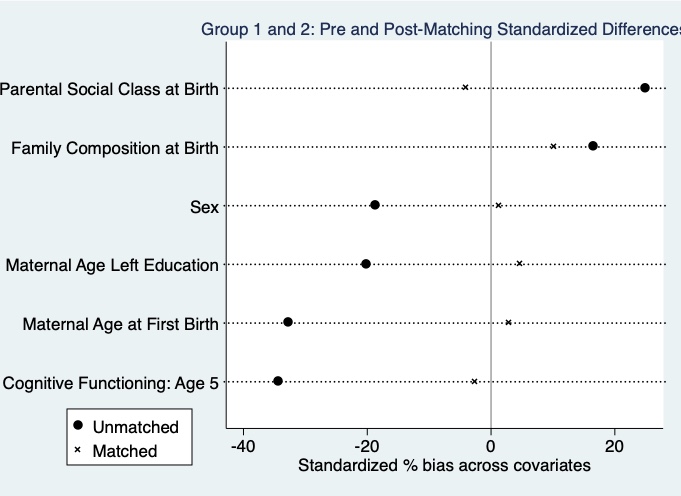 | 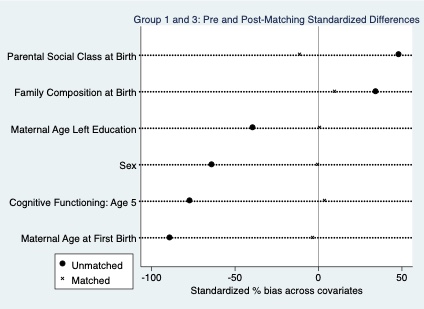 |
| --- | --- |

Note: N for the treatment group in Model 1 (Group 2) was 1,305 and 5,278 for the control group. N for the treatment group in model 2 (Group 3) was 70 and 5,278 for the control group, as 1 participant in the treatment group was off support.

# eFigure 4. Standardised differences across covariates: Pre and post-matching at age 46

| 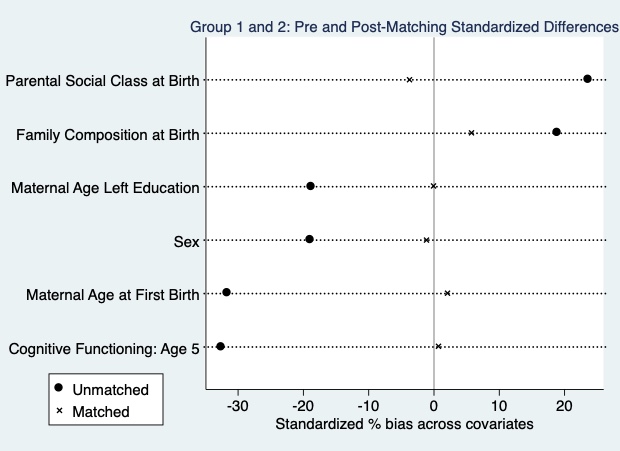 | 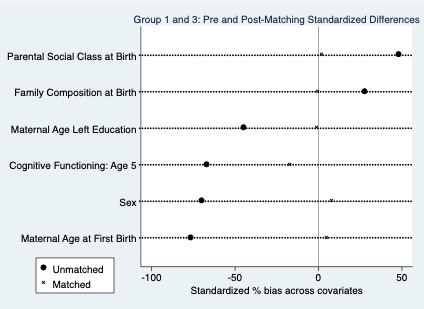 |
| --- | --- |

Note: N for the treatment group in Model 1 (Group 2) was 1,299 and 5,240 for the control group. N for the treatment group in model 2 (Group 3) was 80 and 5,240 for the control group.
